# Lack of Substantial Post-Cessation Weight Increase in Electronic Cigarettes Users

**DOI:** 10.3390/ijerph15040581

**Published:** 2018-03-23

**Authors:** Cristina Russo, Fabio Cibella, Enrico Mondati, Pasquale Caponnetto, Evelise Frazzetto, Massimo Caruso, Grazia Caci, Riccardo Polosa

**Affiliations:** 1 MCAU ARNAS Garibaldi, 95123 Catania, Italy; kristina_russo@yahoo.com; 2 National Research Council of Italy, Institute of Biomedicine and Molecular Immunology, 90100 Palermo, Italy; fabio.cibella@ibim.cnr.it; 3 Institute of Internal and Emergency Medicine, Azienda Ospedaliero-Universitaria “Policlinico-V. Emanuele”, University of Catania, 95123 Catania, Italy; emondati@unict.it (E.M.); evelise.frazzetto@gmail.com (E.F.); mascaru@unict.it (M.C.); grazia.caci15@gmail.com (G.C.); 4 Department of Clinical and Experimental Medicine, University of Catania, 95123 Catania, Italy; p.caponnetto@unict.it; 5 Centro per la Prevenzione e Cura del Tabagismo (CPCT), Azienda Ospedaliero-Universitaria “Policlinico-V. Emanuele”, University of Catania, 95123 Catania, Italy

**Keywords:** smoking cessation, smoking reduction, electronic cigarette, weight gain, tobacco harm reduction

## Abstract

Minimization of post-cessation weight gain in quitters is important, but existing approaches (e.g., antismoking medications) shows only limited success. We investigated changes in body weight in smokers who quit or reduced substantially their cigarette consumption by switching to electronic cigarettes (ECs) use. Body weight and smoking/vaping history were extracted from medical records of smokers and ex-smokers to match three study groups: (1) regular EC users on at least two consecutive follow-up visits; (2) regular smokers (and not using ECs); (3) subjects who reported sustained smoking abstinence after completing a cessation program. Review of their medical records was conducted at two follow-up visits at 6- (F/U 6m) and 12-months (F/U 12m). A total of 86 EC users, 93 regular smokers, and 44 quitters were studied. In the EC users study group, cigarettes/day use decreased from 21.1 at baseline to 1.8 at F/U 12m (*p* < 0.0001). Dual usage was reported by approximately 50% of EC users. Both within factor (time, *p* < 0.0001) and between factor (study groups, *p* < 0.0001) produced significant effect on weight (% change from baseline), with a significant 4.8% weight gain from baseline in the quitters study group at F/U 12m. For the EC users, weight gain at F/U 12m was only 1.5% of baseline. There was no evidence of post-cessation weight increase in those who reduced substantially cigarette consumption by switching to ECs (i.e., dual users) and only modest post-cessation weight increase was reported in exclusive EC users at F/U 12m. By reducing weight gain and tobacco consumption, EC-based interventions may promote an overall improvement in quality of life.

## 1. Introduction

Smoking is the leading cause of preventable premature mortality in the world; total tobacco related deaths are projected to increase from approximately 5 million per year today to over 8 million annually by 2030 [1]. Mortality is mainly due to lung cancer and to the acute fatal complications of ischemic heart disease, stroke and chronic obstructive pulmonary disease (COPD) [2,3].

Quitting smoking is known to reduce the risk of lung cancer, ischemic heart disease, stroke, and COPD [2,3,4]. For those willing to quit, a combination of pharmacotherapy and intensive behavioural intervention for smoking cessation can support quit attempts and can double or triple quit rates [5,6].

However, while stopping smoking results in clear health advantages, it is often accompanied by a significant increase in body weight [7,8,9]. In a large prospective UK study, abstainers gained on average 8.79 kg at eight years, whereas continuing smokers only 2.24 kg [8]. As nicotine (in tobacco cigarettes) is known to suppress appetite and to increase resting metabolic rate [10], weight gain in those who quit smoking is probably due to the combination of a decline in resting energy expenditure at a time when appetite is increased. This knowledge is an important deterrent to many smokers (mainly women) who want to quit [11], and who quote weight loss as a primary reason for smoking [12]. Moreover, smoking cessation-related weight gain may offset some health advantages of giving up smoking. Recent evidence indicates that increase in the prevalence of being overweight and obesity in the US in recent years may be attributed in part to the concurrent drop in smoking [13]. In turn, the rising prevalence of overweight and obesity is a major cause for the growing type 2 diabetes epidemic [14,15].

A Cochrane review that examined the effectiveness of first line antismoking medications (i.e., nicotine replacement therapies, bupropion, and varenicline) in preventing post cessation weight gain [16] showed only modest results, with NRT, bupropion, and varenicline reducing weight gain only by 0.5 kg, 1.1 kg, and 0.4 kg, respectively. Additionally, this modest advantage was lost rapidly after treatment discontinuation. In addition, minimization of post cessation weight gain by general dietary education or exercise programs has not shown consistent results [16]. Consequently, the need for novel and more efficient approaches is undisputable.

Electronic cigarettes (ECs) are battery-operated devices designed to vaporise an e-liquid (a solution mainly consisting of glycerol, propylene glycol, distilled water, and flavourings, which may or may not contain nicotine) by heating an element (most commonly, a metal coil) that generates an inhalable aerosol. The user inhales the aerosol generated by vaporizing the e-liquid in a process commonly referred to as “vaping”. ECs do not contain tobacco, do not create smoke and do not rely on combustion to operate. These consumer products share many similarities with smoking in the behavioural aspect of their use [17]. Users are predominantly smokers who report using them long term as an alternative for conventional cigarettes, to reduce cigarette consumption or quit smoking [18,19,20], to relieve tobacco withdrawal symptoms [21,22], and to continue having a “smoking experience without smoking” [23,24], but with much reduced exposure to toxic emissions [25]. A recent prospective randomized controlled trial has shown that even a mediocre first generation EC (i.e., cigalike) can aid smoking cessation and reduction with long-term quit rates of up to 8.7% in smokers not intending to quit [26]. Much higher success rates have been reported in pilot studies with more advanced second and third generation devices, with an overall quit rate of 36% at six months [27,28]. Nonetheless, the evidence from observational population-based studies on whether ECs might help or hinder smoking cessation is mixed [29,30].

Very little is known about post cessation weight gain after switching to ECs use. Specifically, it is unknown if regular “vaping” (the act of inhaling vapour from ECs) could prevent post cessation weight gain. To address this, we have measured absolute weight changes for up to one year in smokers who quit or reduced substantially their tobacco consumption by switching to ECs and compared these changes to sex- and age-matched smokers who abstained from cigarette smoking after having successfully completed a cessation program.

## 2. Materials and Methods

### 2.1. Study Samples

We conducted a medical records review of patients with cardiorespiratory conditions regularly followed-up at the outpatient clinics of four Italian hospitals. Baseline and follow-up data were extracted from patients’ medical records over a period of approx. 3.5 years (March 2012 to December 2015). Patients reporting regular daily use of ECs (and if at all conventional cigarettes) on at least two consecutive follow-up visits (timed at approx. 6 and 12 months) were eligible for inclusion (*EC users study group*). Details of EC devices and e-liquid nicotine strengths were extracted from their 12-month visit. Patients’ data obtained from the clinic visit immediately preceding the first of the two consecutive follow-up visits—when no EC use was yet reported—was considered as their baseline. Only consecutive follow-up visits timed at approx. 6 and 12 months from baseline were considered for analysis. Datasets from chart review of a second group of age-, sex-matched patients reporting to be regular smokers (and not using ECs) over the same observation period from the same participating clinics were selected as reference (*cigarette smokers study group*). Consecutive follow-up visits were timed as for the EC users study group (at approx. 6 and 12 months from baseline).

We have also collected data from age-, sex-matched smokers in good general health who reported sustained smoking abstinence (for ≥6 months) after successfully completing a cessation program based on licensed medications (nicotine patch, bupropion, or varenicline) in combination with counselling at the local smoking cessation center (Centro per la Prevenzione e Cura del Tabagismo—CPCT) (*quitters study group*). Baseline and follow-up data were extracted from clinic records of patients regularly followed-up at CPCT over a period of approx. 3 years (February 2013 to January 2016). Their baseline measures were obtained before enrolling in the smoking cessation intervention (when they were smoking). For those who achieved documented sustained abstinence, consecutive follow-up visits were timed at approx. 6 and 12 months from baseline.

Distribution of gender and age in the two reference study groups (i.e., cigarette smokers and quitters, respectively) were matched to the cohort of EC users by staff unaware of study design and objectives. They selected clinical records creating sub-samples with similar age distribution (within a 5-year age span) and an M:F ratio close to 2:1. Researchers involved in the study analysis were not involved in patients’ medical management. The study was approved by the ethics review board of the coordinating center (“Policlinico-Vittorio Emanuele Hospitals”) and informed consent was obtained from each patient.

### 2.2. Study Design and Study Assessments

Physicians retrospectively reviewed clinical notes. They extracted patient data from the clinic visit immediately preceding (baseline visit, T_0_) the first of the two follow-ups visits (follow-up visits 1 and 2). In brief, data from the three clinic visits were collected and analysed. Follow-up visits 1 (F/U 6m) and 2 (F/U 12m) were carried out at 6 (±1) and 12 (±2) months after baseline visits, respectively.

Outpatient clinic visits were all scheduled in the morning (before lunch time). At each visit, patients were assessed using a standard approach consisting of clinical examination, review of smoking history, and measurement of body weight and height. Body weight was measured by using a mechanical column scale (Seca, Intermed Srl, San Giuliano Milanese, Italia) with patients removing shoes and heavy clothing. Height measurements were taken at the baseline visit by using a standing scale slide bar. Body mass index (BMI) was computed as weight (kg)/height^2^ (m).

### 2.3. Data Management

All demographics and clinical data for study participants were entered in their medical records at the time of the outpatient visit. For the purposes of this study, patients’ data were later extracted from their medical record and entered into an electronic spread-sheet prior to statistical computation.

### 2.4. Study Outcomes

The primary outcome was the change in body weight from baseline to the final follow-up visit at about 1 year. Secondary outcome was the effect of EC users’ phenotype on weight changes.

### 2.5. E-Cigarettes Users Phenotypes

Subjects in the EC users cohort were classified as exclusive users—individuals who reported exclusive ECs use with no cigarettes smoking (i.e., exclusive vaping with complete self-reported abstinence from tobacco smoking)—or dual users—individuals who reported ECs use in combination with cigarette smoking.

### 2.6. Statistical Analyses

Subjects’ characteristics are presented as means ± standard deviation (SD) for continuous variables and frequency distribution for categorical variables. Differences among groups were investigated by means of one-way analysis of variance (ANOVA) and Kruskal–Wallis test for continuous variables with normal and not normal distribution, respectively. Differences in frequency distribution of categorical variables were evaluated by χ^2^ test.

An Analysis of Variance for Repeated Measurements (RMANOVA) model was built on subjects’ weight to find if belonging to any of the 3 groups affected its values. The variable time (T_0_, F/U 6m, and F/U 12m) entered the model as within factor; the variable group (i.e., EC users, cigarette smokers, and quitters study groups) entered the model as between factor. To evaluate the effect of group on weight changes over the time, taking into account the effect of possible confounding factors, we estimated parameters for % weight change as dependent variable and group, sex, BMI, age, and No. of smoked cigarettes at T_0_ as independent variables, by means of an unique multiple linear regression analysis model. The model was separately built for changes at 6 and 12 months.

The analyses were carried out using Statistical Package for Social Sciences (SPSS Inc., Chicago, IL, USA) for Windows version 20.0 and *p* values < 0.05 were considered significant.

## 3. Results

### 3.1. Patients’ Characteristics

A total of 223 subjects were included in the study and their baseline characteristics are presented in Table 1, separately for each study group. After reviewing 1258 medical records of patients with cardiorespiratory conditions (631 with arterial hypertension; 347 with asthma; 280 with COPD), we identified a total of 86 e (28 F) patients who were regular daily EC users (EC users study group) of which 44 with a diagnosis of arterial hypertension, 18 with asthma and 24 with COPD; after reviewing 315 medical records of patients with cardiorespiratory conditions (179 with arterial hypertension; 72 with asthma; 64 with COPD), we identified a total of 93 (34 F) age-, sex-matched patients who were regular smokers and not using ECs (cigarette smokers study group) of which 51 with a diagnosis of arterial hypertension, 18 with asthma and 24 with COPD; after reviewing 138 records of smokers in good general health attending our smoking cessation clinic, we identified a total of 44 (15 F) age-, sex-matched smokers who reported sustained smoking abstinence (for ≥6 months) after completing a cessation program (quitters study group). There were no significant differences at baseline among study groups regarding sex, age, body weight, BMI, and number of cigarettes smoked per day (Table 1). 

The selected sample provided a power of 73% for detecting significant differences in weight changes between exclusive EC users and quitters, with an alpha value of 0.05. The power rose to 99% for detecting significant differences in weight changes between EC dual users and quitters.

### 3.2. Changes in Smoking Behaviour and Patterns of e-Cigarette Use

At baseline, all subjects smoked an average of >20 conventional cigarettes/day (Table 1). A marked reduction in conventional cigarette use was observed in regular EC users. Their overall mean (range) cigarettes/day use decreased from 21.1 (10–30) at baseline to 2.5 (0–10) at F/U 6m and 1.8 (0–6) at F/U 12m, respectively (*p* < 0.0001). Dual usage was reported by 44/86 (51%) patients at F/U 6m and by 41/86 (48%) patients at F/U 12m, respectively. In dual users, cigarettes/day use was 5.0 (3–10) at F/U 6m and 3.8 (2–6) at F/U 12m. At least 75% reduction from baseline in cigarette/day consumption was reported by 71/86 (83%) patients at F/U 6m and by 84/86 (98%) patients at F/U 12m, respectively.

In the EC user group, EC use ranged from 10–14 months, with 81.4% (70 out of 86) patients using them for more than a year. Details about device type were available from 91.9% (79 out of 86) EC users; 11 users used first generation ECs (i.e., cigalikes), 32 standard refillable second generation ECs (i.e., assorted ECO style products) and 36 more advanced design refillable device. Details about e-liquids nicotine strengths were available from 83.7% (72 out of 86) EC users; all were consuming nicotine-containing e-liquids with 40 users consuming low (4–9 mg/mL), 22 medium nicotine (12–18 mg/mL), and 10 high nicotine strengths (>18 mg/mL).

None of the subjects in the quitters group reported any e-cigarettes use at any time.

### 3.3. Changes in Body Weight

Changes in body weight from baseline are illustrated and reported in Table 2.

Table 2 shows body weight at baseline, at F/U 6m, and at F/U 12m, along with its changes with respect to baseline both in absolute and percent values, separately for each study group. Within the EC users study group, the values relevant to exclusive EC users and dual users are also shown.

The RMANOVA model showed that both within factor (time, *p* < 0.0001) and between factor (study groups, *p* < 0.0001) produced significant effect on weight (%change from baseline), but that only in the quitters study group was a significant effect still evident at F/U 12m, with an average weight gain from baseline of 4.8% ± 4.5. At F/U 12m, the average weight gain for the EC users was only 1.5% ± 4.1. 

**Figure 1:**
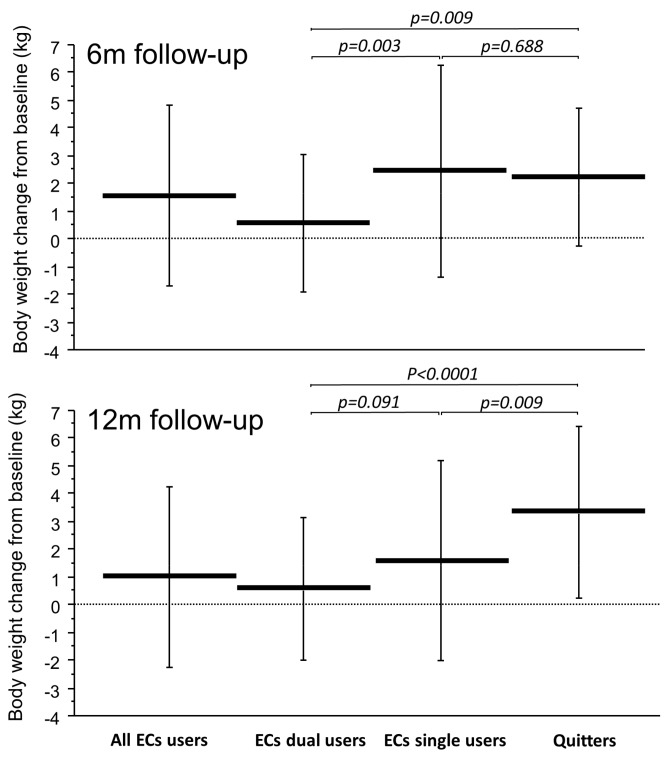
Absolute body weight changes from baseline at 6 (upper panel) and at 12 (lower panel) months follow-up separately for all EC users, EC dual users, exclusive EC users (i.e., single users), and Quitters. Horizontal and vertical lines indicate means and standard deviations, respectively. *p*-values were computed by means of one-way Analysis of Variance and Fisher’s Least Significant Difference.

In Figure 1, the absolute weight changes from baseline at F/U 6 m and 12 m are illustrated separately for Dual and exclusive EC users and compared to those of quitters. At F/U 6m, exclusive EC users had similar weight increase to quitters (2.5 ± 3.7 kg vs. 2.3 ± 2.5 kg; *p* = 0.688) and were significantly different from that of dual users (2.5 ± 3.7 kg vs. 0.6 ± 2.5 kg; *p* = 0.003). However, exclusive EC users’ weight increase at F/U 12m was significantly lower than quitters (1.6 ± 3.6 kg vs. 3.4 ± 3.0 kg; *p* = 0.009) and not statistically different from that of dual users (1.6 ± 3.6 kg vs. 0.8 ± 2.9 kg; *p* = 0.091).

Table 3 presents the results of the multiple linear regression analysis models for percent weight change as dependent variable and group (taking separately into account the exclusive EC users and dual users), sex, age, BMI, and number of cigarettes smoked at T_0_ as independent variables. The model was separately built for evaluating changes at 6 and 12 months. At 6m follow-up, significant B coefficients were found for both quitters (2.79) and exclusive EC users (2.91) study groups with respect to the regular smokers study group. At 12m follow-up, the slope for quitters study group was 4.13 while the slope relevant to the exclusive EC users was no longer significant and about one-third (1.27) with respect to quitters. The slope of EC dual users was never significant with respect to the regular smokers study group.

## 4. Discussion

Stopping smoking is known to result in a significant increase in body weight [7,8,9] and current approaches aimed at minimizing post cessation weight gain have been shown to be largely ineffective [16]. Here, we show a lack of substantial weight increase in smokers who quit or dramatically reduced their tobacco consumption by vaping regularly. These findings are of great significance bearing in mind the negative health impact that any increase in body weight gain has on cardiovascular diseases, metabolic conditions, and some cancers [31]. Moreover, lack of weight gain by switching to regular ECs use may be an important incentive to quit for those smokers who are concerned about cessation-related weight gain [12,32,33].

As expected, this study confirms previous observations that tobacco smoking helps prevent weight gain [34,35], with no significant increase in weight being observed in the cohort of smokers who continued to smoke. On the other hand, smokers who reported sustained smoking abstinence after completing a cessation program had a significant increase in body weight; on average, they gained 2.3 and 3.4 kg of weight at 6 and 12 months, respectively. These findings are consistent with previously published data [7,8,9,36,37] and with a meta-analysis of 62 prospective studies [38].

An important finding of the present study, however, is that regular use of ECs resulted in reduced weight gain in those who quit or reduced substantially their tobacco consumption. In our study, EC users gained on average 1.6 kg and 1.1 kg at 6 months and 12 months, respectively. Thus, the weight gain measured after switching to ECs was smaller than in smokers who successfully quit smoking (and not using ECs), and particularly at 12 months. Although this contrasts with smoking cessation trials of licensed pharmacotherapies where substantial weight gain has been reported systematically [16], the findings of the present study are consistent with those of a prospective randomized controlled trial with early design cigalikes, which did not report significant long-term weight gain when compared with continuing smokers and reducers [39]. Our current retrospective study differs from previous work [39] in that it explores the effect of more advanced devices in a much larger population of experienced users. Moreover, in a clinical trial of 387 adult smokers randomised to either switching to ECs (and substantially reducing tobacco cigarette consumption to below 2 cig/day throughout the study) or continuing to smoke their own tobacco cigarette brand for a total of 12 weeks, body weight remained stable in both study groups [40]. 

It is unlikely that the observed lack of substantial post-cessation weight increase in our EC users is due to specific confounders. Bearing in mind that women tend to gain more weight than men after smoking cessation [41,42], if the proportion of women who quit in a given study is substantially different from men, the level of post-cessation weight gain will probably reflect the relative contribution of the female population to the overall study sample. However, this was not the case in our study because female participation was balanced across the three study groups. In any case, the results of the multiple linear regression analysis for weight change showed no significant effect for gender. In addition, no effect was detected for age, baseline BMI, and smoking intensity (i.e., number of cigarettes reported at baseline). Being weight-conscious at time of smoking cessation (or switching) may be another important confounder of the relation between smoking and change in body weight, but this was not measured. Smokers attending smoking cessation programs are generally given tailored instructions to prevent post-cessation weight gain that focus on careful control of eating behaviours. In any case, this would have underestimated (rather than overestimated) the increase in body weight observed in the quitters study group.

When weight changes were analysed separately for Dual and exclusive EC users, there were notable differences. Whereas exclusive EC users had similar weight increase to quitters at six months, their weight gain was no longer evident at 12 months being reduced by more than 50% compared to quitters. Dual users, instead, consistently gained less weight than quitters at both time points. Both findings were unexpected. For the exclusive EC users, it was surprising to observe such a dichotomy. It is difficult to provide sufficient explanation to clarify the complex gain and drop in weight among EC users over time. We speculated that the higher prevalence of attendance at the first follow-up for the exclusive EC users study group in winter months, when more calorically dense food is consumed and individuals are generally less active, could have been a contributing factor [43,44]. For the dual users, it was equally surprising to observe such an important limitation of their weight gain considering that their cigarette consumption was so low (on average 2–3 cig/day) to have enabled the compensatory effect of smoking. It is possible that, when smoking is restricted during main meals, even a few cigarettes can interfere with eating patterns. Unfortunately, we did not measure food intake in this study. In addition, the complex gain and drop in weight among EC users over time could have been due to graduation from less efficient to more efficient device types; unfortunately, this specific information was not available in the medical record. Further speculation includes the possibility that (1) intentional weight loss at a later stage can be associated with those exclusive EC users who started to embrace a healthier lifestyle; and (2) regular exposure to some flavourings in the e-liquid aerosol may induce satiation enhancement, thus reversing post-cessation weight gain with time.

Although our data do not allow us to specify the bio-behavioral processes underlying the reported changes in body weight, lack of substantial post-cessation weight increase in EC users is an important finding and requires explanation. Because ECs replicate many of the sensory characteristics and rituals associated with smoking, these positive findings could be explained by the great compensatory effect of ECs at both physical and behavioral level [45,46].

The mechanisms posited to underlie post cessation weight gain include a decline in resting energy expenditure and an increase in food intake due to the withdrawal of nicotine [10]. It is possible that substitution of conventional cigarettes with ECs minimizes post-cessation weight gain by preserving or increasing levels of nicotine in the body; 86% of the EC users in our study used second and third generation ECs that have been reported to deliver nicotine at levels close to those obtained from conventional cigarettes [47,48].

Another possible mechanism is that substitution of conventional cigarettes with devices that mimic the hand-to-mouth action of smoking might have provided a coping mechanism for the compulsive eating that arises after cigarette withdrawal [49]. Indeed, even earlier first generation ECs that were shown to be unable to deliver sufficient nicotine levels could nonetheless substantially reduce post cessation weight gain [41]. Despite allowing considerably higher nicotine delivery, the nicotine patch is much less effective in reducing post cessation weight gain compared to nicotine chewing gum [16]. This has been associated with the faster delivery of nicotine by gums, but other explanations are possible (e.g., the act of chewing may help coping with compulsive eating).

There are some limitations in our study. Firstly, this is a relatively undersized retrospective study, and results must be interpreted with prudence. Despite the limited number of subjects, results of the study’s primary outcome were significant and consistent. Standard concerns associated with retrospective studies (including variance in the quality of information recorded by medical professionals and difficulty in establishing a causal relationship) also need consideration. Secondly, no objective assessment of smoking abstinence was performed and cigarette consumption was self-reported. However, the self-reported number of cigarettes smoked per day in studies of this type is not subjected to the kind of biases observed in clinical trials where there is the tendency to claim abstinence [50]. Moreover, similar beneficial effects were also reported in dual users (i.e., smoking reducers) and therefore objective measures of abstinence are unlikely to be of great importance. Additionally, confounding factors, which may have had an influence on weight changes (e.g., weight concern, dietary habits, recreational exercise), were not assessed. Last but not least, smokers with cardiopulmonary conditions are not directly comparable with smokers in good general health, and this may have a significant impact on the study’s findings. However, there is no evidence that ambulatory patients with mild-moderate cardiorespiratory conditions would behave differently from age- sex-matched healthy smokers from a metabolic standpoint as well as in terms of food consumption. For example, a study has shown that patients with mild COPD will put up same weight gain as healthy smokers after smoking cessation [51]. In any case, patients with cardiopulmonary conditions are likely to be more sedentary than age- and sex-matched healthy smokers and therefore prone to gain extra weight, thus underestimating (rather than overestimating) the findings of our study. Therefore, we do not feel that comparing smokers with cardiopulmonary conditions with age- sex-matched smokers in good general health might have meaningfully clouded the study’s findings. 

## 5. Conclusions

More research is necessary to better delineate the risk/benefit ratio of e-cigarettes [52,53].

Within the study limitations, EC use may help smokers attenuate cigarette consumption or remain abstinent, as well as reduce their post-cessation weight increase. The potential role of the e-vapour category for harm minimization in relation to tobacco and/or food abuse requires confirmation from larger prospective studies. Moreover, the observed lack of post-cessation weight gain in those who reduced substantially cigarette consumption by switching to ECs (i.e., dual users) is an interesting finding and calls for further research investigating the role of nicotine in weight control. Meanwhile, these preliminary findings should be communicated to smokers and particularly to weight-conscious smokers intending to quit.

By combining substantial reduction of smoking with prevention of post-cessation weight gain, EC-based interventions may promote an overall improvement in quality of life. Considering that the negative effects of weight increase could overshadow the health benefits of smoking abstinence [54,55,56], it is important to stimulate more research in this area.

## Figures and Tables

**Table 1:** Subjects’ characteristics at baseline (T0).

Parameters	EC Users Study Group N = 86	Cigarette Smokers Study Group N = 93	Quitters Study Group N = 44
Sex (No., M/F)	58/28	59/34	29/15
Age (Years, mean ± SD)	54.2 ± 12.6	54.3 ± 11.7	53.3 ± 12.7
Body weight (kg, mean ± SD)	72.7 ± 11.9	72.5 ± 12.0	74.5 ± 8.9
BMI (kg/m^2^, mean ± SD)	26.0 ± 3.0	26.3 ± 3.3	26.7 ± 2.6
Cig/day (cig/day, mean ± SD)	21.1 ± 5.0	20.5 ± 4.7	20.2 ± 3.9

At baseline, the distribution of both categorical and continuous variables was not statistically different among study groups. ECs: electronic cigarettes; SD: standard deviation; BMI: body mass index.

**Table 2:** Body weight (BW) at baseline (BW T0), 6 (BW 6m) and 12 months (BW 12m) and associated changes both in percent values and absolute differences.

	BW T_0_ (kg, Mean ± SD)	BW 6m (kg, Mean ± SD)	BW 6m * (%T_0_, Mean ± SD)	BW 6m ** (diff T_0_, kg, Mean ± SD)	BW 12m (kg, Mean ± SD)	BW 12m * (%T_0_, Mean ± SD)	BW 12m ** (diff T_0_, kg, Mean ± SD)
Cigarette smokers study group N = 93	72.5 ± 12.0	72.7 ± 11.8	0.5 ± 2.6	0.3 ± 1.8	72.9 ± 11.9	0.7 ± 2.9	0.5 ± 2.1
EC users study group N = 86	72.7 ± 11.9	74.2 ± 12.6	2.2 ± 4.4 (*p* = 0.387) ^†^	1.6 ± 3.3	73.8 ± 12.4	1.5 ± 4.1 (*p* = 0.627) ^†^	1.1 ± 3.2
Exclusive EC users ^§^ Dual users	72.1 ± 11.7 73.2 ± 12.2	74.7 ± 12.8 73.8 ± 12.4	3.5 ± 4.9 0.9 ± 3.4	2.5 ± 3.7 0.6 ± 2.5	72.6 ± 13.2 75.0 ± 11.5	2.2 ± 4.6 0.7 ± 3.5	1.6 ± 3.6 0.8 ± 2.9
Quitters study group N = 44	74.5 ± 8.9	76.8 ± 8.6	3.2 ± 3.6 (*p* = 0.048) ^†^	2.3 ± 2.5	77.9 ± 8.4	4.8 ± 4.5 (*p* = 0.019) ^†^	3.4 ± 3.0

* Changes from baseline in % values; ** Changes from baseline in absolute values (kg). ^§^ At 6 months N = 42; at 12 months N = 45. At 6 months N = 44; at 12 months N = 41. ^†^ vs. cigarette smoker study group. ECs: electronic cigarettes; SD: standard deviation.

**Table 3:** Parameters estimated by multiple linear regression analysis models for percent weight change at 6 and 12 months as dependent variable and group, sex, age, BMI at baseline, and number of cigarettes smoked at baseline as independent variables.

Variables	Weight Change at 6 Months			Weight Change at 12 Months		
	B	95% CI	*p*	B	95% CI	*p*
Quitters study group *	2.794	1.549/4.039	**<0.0001**	4.127	2.780/5.474	**<0.0001**
EC users study group-exclusive EC users *	2.908	1.639/4.176	**<0.0001**	1.274	−0.034/2.714	0.054
EC users study group-dual users *	0.419	−0.830/1.669	0.509	0.076	−1.309/1.460	0.914
Sex **	0.630	−0.339/1.599	0.201	0.983	−0.066/2.031	0.066
Age (years)	−0.024	−0.062/0.014	0.212	−0.019	−0.060/0.0122	0.357
BMI at T_0_ (kg/m^2^)	−0.088	−0.239/0.063	0.252	−0.126	−0.289/0.038	0.131
Cig/day at T_0_ (No.)	0.054	−0.045/0.152	0.287	0.043	−0.064/0.149	0.434

* Reference: Cigarette smokers study group; ** reference: Males. ECs: electronic cigarettes; CI: confidence intervals.

## References

[1] US Department of Health and Human Services (2014). The Health Consequences of Smoking—50 Years of Progress: A Report of the Surgeon General.

[2] World Health Organization (2008). WHO Report on the Global Tobacco Epidemic, 2008—The MPOWER Package.

[3] Doll R., Peto R., Boreham J., Sutherland I. (2004). Mortality in relation to smoking: 50 years’ observations on male British doctors. BMJ.

[4] U.S. Department of Health and Human Services (1990). The Health Benefits of Smoking Cessation: A Report of the Surgeon General.

[5] Polosa R., Benowitz N.L. (2011). Treatment of nicotine addiction: Present therapeutic options and pipeline developments. Trends. Pharmacol. Sci..

[6] Stead L.F., Koilpillai P., Fanshawe T.R., Lancaster T., Stead L.F. (2016). Combined pharmacotherapy and behavioural interventions for smoking cessation. Cochrane Database of Systematic Reviews.

[7] Klesges R.C., Meyers A.W., Klesges L.M., La Vasque M.E. (1989). Smoking, body weight, and their effects on smoking behavior: A comprehensive review of the literature. Psychol. Bull..

[8] Lycett D., Munafò M., Johnstone E., Murphy M., Aveyard P. (2011). Associations between weight change over 8 years and baseline body mass index in a cohort of continuing and quitting smokers. Addiction.

[9] Eisenberg D., Quinn B.C. (2006). Estimating the effect of smoking cessation on weight gain: An instrumental variable approach. Health Serv. Res..

[10] Zoli M., Picciotto M.R. (2012). Nicotinic Regulation of Energy Homeostasis. Nicot. Tob. Res..

[11] Brouwer R.J.N., Pomerleau C.S. (2000). “Prequit attrition” among weight-concerned women smokers. Eat. Behav..

[12] Rosenthal L., Carroll-Scott A., Earnshaw V.A., Sackey N., O’Malley S.S., Santilli A., Ickovics J.R. (2013). Targeting cessation: Understanding barriers and motivations to quitting among urban adult daily tobacco smokers. Addict. Behav..

[13] Courtemanche C., Tchernis R., Ukert B. (2018). The Effect of Smoking on Obesity: Evidence from a Randomized Trial. J. Health Econ..

[14] Davey Smith G., Bracha Y., Svendsen K.H., Neaton J.D., Haffner S.M., Kuller L.H. (2005). Multiple Risk Factor Intervention Trial Research Group Incidence of type 2 diabetes in the randomized multiple risk factor intervention trial. Ann. Intern. Med..

[15] Yeh H.C., Duncan B.B., Schmidt M.I., Wang N.Y., Brancati F.L. (2010). Smoking, Smoking Cessation, and Risk for Type 2 Diabetes Mellitus. Ann. Intern. Med..

[16] Farley A.C., Hajek P., Lycett D., Aveyard P., Aveyard P. (2016). Interventions for preventing weight gain after smoking cessation. Cochrane Database of Systematic Reviews.

[17] Caponnetto P., Russo C., Bruno C.M., Alamo A., Amaradio M.D., Polosa R. (2015). Electronic cigarette: A possible substitute for cigarette dependence. Monaldi Arch. Chest Dis..

[18] Farsalinos K.E., Romagna G., Tsiapras D., Kyrzopoulos S., Voudris V. (2014). Characteristics, perceived side effects and benefits of electronic cigarette use: A worldwide survey of more than 19,000 consumers. Int. J. Environ. Res. Public Health.

[19] Gallus S., Lugo A., Pacifici R., Pichini S., Colombo P., Garattini S., La Vecchia C. (2014). E-Cigarette Awareness, Use, and Harm Perceptions in Italy: A National Representative Survey. Nicot. Tob. Res..

[20] Pechacek T.F., Nayak P., Gregory K.R., Weaver S.R., Eriksen M.P. (2016). The Potential That Electronic Nicotine Delivery Systems Can be a Disruptive Technology: Results from a National Survey. Nicot. Tob. Res..

[21] Dawkins L., Kimber C., Puwanesarasa Y., Soar K. (2015). First-versus second-generation electronic cigarettes: Predictors of choice and effects on urge to smoke and withdrawal symptoms. Addiction.

[22] Etter J.F. (2017). Gateway effects and electronic cigarettes. Addiction.

[23] Kistler C., Crutchfield T., Sutfin E., Ranney L., Berman M., Zarkin G., Goldstein A. (2017). Consumers’ Preferences for Electronic Nicotine Delivery System Product Features: A Structured Content Analysis. Int. J. Environ. Res. Public Health.

[24] Soule E.K., Maloney S.F., Guy M.C., Eissenberg T., Fagan P. (2017). User identified positive outcome expectancies of electronic cigarette use: A concept mapping study. Psychol. Addict. Behav..

[25] Farsalinos K.E., Polosa R. (2014). Safety evaluation and risk assessment of electronic cigarettes as tobacco cigarette substitutes: A systematic review. Ther. Adv. Drug Saf..

[26] Caponnetto P., Campagna D., Cibella F., Morjaria J.B., Caruso M., Russo C., Polosa R. (2013). EffiCiency and Safety of an eLectronic cigAreTte (ECLAT) as tobacco cigarettes substitute: A prospective 12-month randomized control design study. PLoS ONE.

[27] Polosa R., Caponnetto P., Maglia M., Morjaria J.B., Russo C. (2014). Success rates with nicotine personal vaporizers: A prospective 6-month pilot study of smokers not intending to quit. BMC Public Health.

[28] Adriaens K., Van Gucht D., Declerk P., Baeyens F. (2014). Effectiveness of the Electronic Cigarette: An Eight-Week Flemish Study with Six-Month Follow-up on Smoking Reduction, Craving and Experienced Benefits and Complaints. Int. J. Environ. Res. Public Health.

[29] Zhu S.H., Zhuang Y.L., Wong S., Cummins S.E., Tedeschi G.J. (2017). E-cigarette use and associated changes in population smoking cessation: Evidence from US current population surveys. BMJ.

[30] Doran N., Brikmanis K., Petersen A., Delucchi K., Al-Delaimy W.K., Luczak S., Myers M., Strong D. (2017). Does e-cigarette use predict cigarette escalation? A longitudinal study of young adult non-daily smokers. Prev. Med..

[31] GBD 2015 Obesity Collaborators (2017). Health Effects of Overweight and Obesity in 195 Countries over 25 Years. N. Engl. J. Med..

[32] Clark M.M., Hurt R.D., Croghan I.T., Patten C.A., Novotny P., Sloan J.A., Dakhil S.R., Croghan G.A., Wos E.J., Rowland K.M. (2006). The prevalence of weight concerns in a smoking abstinence clinical trial. Addict. Behav..

[33] Tuovinen E.-L., Saarni S.E., Kinnunen T.H., Haukkala A., Jousilahti P., Patja K., Kaprio J., Korhonen T. (2015). Associations of Weight Concerns With Self-Efficacy and Motivation to Quit Smoking: A Population-Based Study Among Finnish Daily Smokers. Nicot. Tob. Res..

[34] Shimokata H., Muller D.C., Andres R. (1989). Studies in the Distribution of Body Fat. JAMA.

[35] Klesges R.C., Ward K.D., Ray J.W., Cutter G., Jacobs D.R., Wagenknecht L.E. (1998). The prospective relationships between smoking and weight in a young, biracial cohort: The Coronary Artery Risk Development in Young Adults Study. J. Consult. Clin. Psychol..

[36] O’Hara P., Connett J.E., Lee W.W., Nides M., Murray R., Wise R. (1998). Early and late weight gain following smoking cessation in the Lung Health Study. Am. J. Epidemiol..

[37] Pistelli F., Aquilini F., Carrozzi L. (2016). Weight Gain after Smoking Cessation. Monaldi Arch. Chest Dis..

[38] Aubin H.-J., Farley A., Lycett D., Lahmek P., Aveyard P. (2012). Weight gain in smokers after quitting cigarettes: Meta-analysis. BMJ.

[39] Russo C., Cibella F., Caponnetto P., Campagna D., Maglia M., Frazzetto E., Mondati E., Caruso M., Polosa R. (2016). Evaluation of Post Cessation Weight Gain in a 1-Year Randomized Smoking Cessation Trial of Electronic Cigarettes. Sci. Rep..

[40] Cravo A.S., Bush J., Sharma G., Savioz R., Martin C., Craige S., Walele T. (2016). A randomised, parallel group study to evaluate the safety profile of an electronic vapour product over 12 weeks. Regul. Toxicol. Pharmacol..

[41] Williamson D.F., Madans J., Anda R.F., Kleinman J.C., Giovino G.A., Byers T. (1991). Smoking Cessation and Severity of Weight Gain in a National Cohort. N. Engl. J. Med..

[42] Flegal K.M., Troiano R.P., Pamuk E.R., Kuczmarski R.J., Campbell S.M. (1995). The Influence of Smoking Cessation on the Prevalence of Overweight in the United States. N. Engl. J. Med..

[43] Yanovski J.A., Yanovski S.Z., Sovik K.N., Nguyen T.T., O’Neil P.M., Sebring N.G. (2000). A prospective study of holiday weight gain. N. Engl. J. Med..

[44] Hull H.R., Radley D., Dinger M.K., Fields D.A. (2006). The effect of the Thanksgiving holiday on weight gain. Nutr. J..

[45] Caponnetto P., Cibella F., Mancuso S., Campagna D., Arcidiacono G., Polosa R. (2011). Effect of a nicotine-free inhalator as part of a 605 smoking-cessation programme. Eur. Respir. J..

[46] Wills T.A., Sargent J.D., Gibbons F.X., Pagano I., Schweitzer R. (2016). E-cigarette use is differentially related to smoking onset among lower risk adolescents. Tob. Control.

[47] Farsalinos K.E., Spyrou A., Tsimopoulou K., Stefopoulos C., Romagna G., Voudris V. (2014). Nicotine absorption from electronic cigarette use: Comparison between first and new-generation devices. Sci. Rep..

[48] Fearon I.M., Eldridge A., Gale N., Shepperd C.J., McEwan M., Camacho O.M., Nides M., McAdam K., Proctor C.J. (2017). E-cigarette Nicotine Delivery: Data and Learnings from Pharmacokinetic Studies. Am. J. Health Behav..

[49] Pomerleau C.S., Ehrlich E., Tate J.C., Marks J.L., Flessland K.A., Pomerleau O.F. (1993). The female weight-control smoker: A profile. J. Subst. Abuse.

[50] Wong S.L., Shields M., Leatherdale S., Malaison E., Hammond D. (2012). Assessment of validity of self-reported smoking status. Health Rep..

[51] Nides M., Rand C., Dolce J., Murray R., O’hara P., Voelker H., Connett J. (1994). Weight gain as a function of smoking cessation and 2-mg nicotine gum use among middle-aged smokers with mild lung impairment in the first 2 years of the Lung Health Study. Health Psychol..

[52] National Academies of Sciences, Engineering, and Medicine (2018). Public Health Consequences of E-Cigarettes.

[53] Evidence Review of E-Cigarettes and Heated Tobacco Products 2018: Executive Summary. https://www.gov.uk/government/publications/e-cigarettes-and-heated-tobacco-products-evidence-review/evidence-review-of-e-cigarettes-and-heated-tobacco-products-2018-executive-summary.

[54] Chinn S., Jarvis D., Melotti R., Luczynska C., Ackermann-Liebrich U., Antó J.M., Cerveri I., de Marco R., Gislason T., Heinrich J. (1998). Smoking cessation, lung function, and weight gain: A follow-up study. Lancet.

[55] Stewart S.T., Cutler D.M., Rosen A.B. (2009). Forecasting the Effects of Obesity and Smoking on U.S. Life Expectancy. N. Engl. J. Med..

[56] Yoon C., Goh E., Park S.M., Cho B. (2010). Effects of smoking cessation and weight gain on cardiovascular disease risk factors in Asian male population. Atherosclerosis.

